# A high mutation rate of immunoglobulin heavy chain variable region gene associates with a poor survival and chemotherapy response of mantle cell lymphoma patients

**DOI:** 10.1097/MD.0000000000015811

**Published:** 2019-05-31

**Authors:** Xianqian Li, Ning Wu, Bin Li

**Affiliations:** a Clinical Laboratory, Shanghai Yangpu District Psychiatric Hospital; b Department of Hematology; c Department of Pathology, Shanghai Xuhui Central Hospital, Shanghai, China.

**Keywords:** DNA sequencing, Immunoglobulin heavy chain variable gene, mantle cell lymphoma, overall survival, variable region

## Abstract

Immunoglobulin heavy chain variable region (*IGHV*) gene mutation status is a biomarker for the prognosis of chronic lymphocytic leukemia, whether it is associated with the diagnosis, staging, and prognosis of patients with mantle cell lymphoma (MCL) remains to be determined.

The *IGHV* gene mutations of 52 MCL patients were determined by DNA sequencing and compared with published *IGHV* germline sequences.

DNA sequence alignment of *IGHV* variable regions with published *IGHV* germline sequences showed that the coincidence rate was 94% to 100%. Ten cases (21%) were significantly mutated with the rate of 96.9% to 94.0%. The overall survival time of patients was negatively correlated with the degree of *IGHV* gene mutation. Further survival analysis with log-rank test demonstrated that the patients with significant *IGHV* gene mutations showed a trend towards poor survival.

The mutation rate of the *IGHV* variant region may be determined to assess the prognosis and overall survival time of MCL patients.

## 1 Introduction

Mantle cell lymphoma (MCL) is a specific type of highly invasive B cell malignant lymphoma, which accounts for 2% to 10% of non-Hodgkin lymphoma cases.^[1–3]^ MCL originates from the inner layer of the follicular tissue without antigen-stimulated CD5+, CD23 negative cells.^[4,5]^ MCL preferentially occurs in older men, with an average onset age of approximately 60 years old. Primary MCL is mainly found in the lymph nodes, is characterized by systemic lymphadenopathy and involves the liver, spleen, bone marrow, and peripheral blood. Compared with other mature B cell lymphoid neoplasms, such as chronic lymphocytic leukemia and small lymphocytic lymphoma, MCL is aggressive and often presents as stage III or IV. Modern radiotherapy and chemotherapy have improved the survival of patients with MCL, however the prognosis of MCL is still poor with an average survival time of 4 to 5 years. Thus, the correct diagnosis of MCL, as distinguished from other small B-cell lymphomas, is very important for clinical treatment options and patient prognosis.^[6,7]^

In the past, a diagnosis of MCL has mainly relied on the lymph nodes, tissue biopsies, and bone marrow or peripheral blood immunophenotype. Histologically, MCL belongs to the class of small B cell malignant lymphoma, which consists of small to medium large tumorous lymphocytes with single formality. A typical case of MCL can be morphologically distinguished from other small B cell malignant lymphomas. However, it is difficult to diagnose atypical MCL or MCL from poor quality tissue samples through morphological observation alone, therefore, it is necessary to find a reliable auxiliary differential diagnosis method for MCL.^[6–8]^ With the rapid development of molecular biology, especially next generation sequencing, it is currently possible to assist in the diagnosis and assessment of prognosis of MCL at the genetic level.^[9–11]^

It has been reported that an immunoglobulin heavy chain variable region (*IGHV*) gene mutation status is a biomarker for the prognosis of chronic lymphocytic leukemia patients after treatment with chemotherapeutics and molecular targeted drugs.^[12,13]^ Recent studies have demonstrated that the mutation rate of the *IGHV* gene in MCL tumor cells may be related to the progression of the disease and affect the efficacy of chemotherapy and radiotherapy in mouse models.^[10]^ However, it remains to be determined whether the mutation rate of the *IGHV* gene is a potential biomarker that can be used in clinical MCL diagnosis, staging, prognosis, and follow-up. In the present study, we collected 52 paraffin-embedded tissues from patients with MCL. The *IGHV* gene fragment was amplified by polymerase chain reaction (PCR) and the DNA sequences of the variable region of the *IGHV* gene were determined through DNA sequencing, followed by the evaluation of the mutation rate of the *IGHV* gene in the diagnosis, staging, treatment monitoring, and prognosis of MCL. Our results reveal that the mutation rate of the variable region of the *IGHV* gene was correlated with clinical stage VI, lymph node enlargement, chemotherapy response, and 5-year survival in MCL patients.

## 2 Materials and methods

### 2.1 Patients

Between January 2008 and December 2015, a total of 52 paraffin-embedded tumor tissue samples were collected from MCL patients at the Department of Pathology of Shanghai Xuhui Central Hospital (Shanghai, China). The diagnosis of MCL was performed according to the 2008 World Health Organization (WHO) classification criteria. There was no significant difference in age or sex between the groups of patients from whom the tissue was taken. This study was approved by the ethics committee of the Shanghai Xuhui Central Hospital in line with the Declaration of Helsinki. Written informed consent was obtained from each patient.

### 2.2 Genomic DNA extraction

For each patient, approximately 6 to 8 slices of paraffin embedded tissue, with a thickness of 10 mm, were incubated in xylene for 5 minutes, followed by centrifugation at 13,000 × *g* for 3 minutes. The tissue pellet was combined with tissue lysis buffer of GentraPuregene Tissue kit (Qiagen, Shanghai) supplemented with proteinase K and incubated at 55 °C overnight, followed by the addition of RNase A solution and incubation at 37 °C for 15 minutes. After cooling in an ice-water bath for 1 minute, the mixture was combined with a protein precipitation solution and centrifuged at 13,000 × *g* for 3 minutes. The supernatant was transferred to a new tube, combined with an equal volume of isopropanol and centrifuged at 13,000 × *g* for 5 minutes. The pellet at the bottom of the tube was washed with 70% ethanol, centrifuged at 13,000 × *g* for 5 minutes, dried at room temperature for 5 minutes, dissolved in 50 μL DNA solution, and incubated at 65 °C for approximately 1 hour. The DNA concentration and the A260/A280 ratio were determined using a nucleic acid protein detector (Eppendorf, Shanghai, P.R.China) and the DNA samples were stored at –80 °C until use.

### 2.3 PCR amplification and DNA sequencing

Directed PCR amplification and DNA sequencing of the *IGHV* variable regions were performed by an immunoglobulin heavy chain (IGH) somatic hypermutationassay V2.0 (InVivoScribe Technologies, California), according to the manufacturer's instructions. PCR was performed using TaqPCR master mix (AmpliTaqGold DNA Polymerase Applied Biosystems, ThermoFisher, Shanghai, P.R.China) with approximately 500 ng of extracted genomic DNA. The PCR conditions were as follows: pre-denaturation at 95 °C for 7 minutes; 34 cycles of denaturation (95 °C for 45 seconds), annealing (60 °C for 45 seconds) and extension (72 °C for 90 seconds); final extension at 72 °C for 10 minutes. Ten microliters of PCR amplification products were detected by 2% agarose gel electrophoresis. Positive bands with around 310 bp were subjected to DNA gel extraction (Qiagen, Shanghai). The purified DNA was used as a template for bi-directional DNA sequencing using the primers contained in the IGH somatic hypermutation assay V2.0 kit. DNA sequencing was performed using the ABI3500DX gene sequencer (ABI).

### 2.4 *IGHV* gene sequence analysis

The *IGHV* gene sequence analysis was performed using the online IgBLAST alignment test and the published *IGHV* gene germline sequences (http://www.ncbi.nlm.nih.gov/igblast/). The percentage of difference (%) was calculated.

### 2.5 Statistical analysis

All statistical analyses were performed using SAS verion 9.2 (SAS Institute, Cary, NC). For continuous variables, the median and range were calculated. A chi-squared test was performed in a univariate analysis for the relationship between *IGHV* gene mutation rate and patient clinic factors. Multivariate analysis was evaluated by logistic regression analysis. The Cox proportional hazards regression model was performed for univariate and multivariate survival analysis. Overall survival rates were estimated using the Kaplan–Meier method and compared using the log-rank test. *P*-values of <.05 were considered to be statistically significant in all of the statistical analyses.

## 3 Results

### 3.1 Amplification of *IGHV* gene in paraffin-embedded mantle cell lymphomas

**Figure 1:**
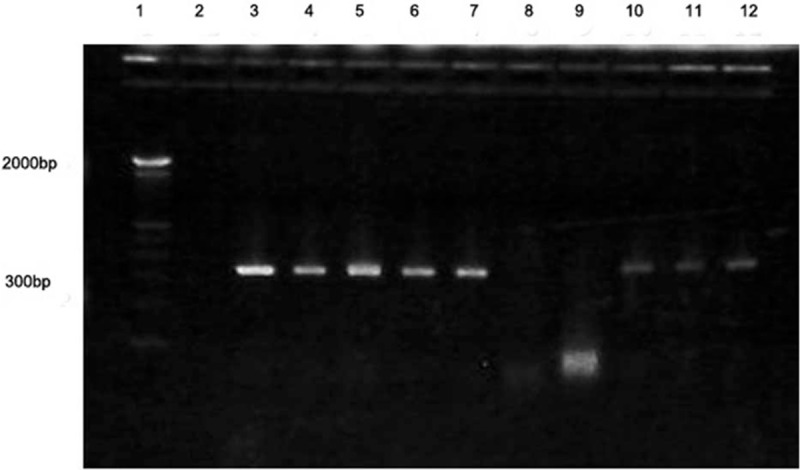
PCR amplification of *IGHV* gene in paraffin-embedded tissue samples from mantle cell lymphoma. Lane 1: marker; Lane 2: blank control; Lane 3: positive control; Lanes 4–7 and 10–12: MCL samples; Lanes 8–9: tumor adjacent tissue samples. *IGHV* = immunoglobulin heavy chain variable region, MCL = mantle cell lymphoma, PCR = polymerase chain reaction.

Of all of the 52 samples, the extracted DNA concentration was approximately 100 to 400 μg/mL with an A260/A280 ratio between 1.7 and 2.0. In the *IGHV* gene PCR-directed amplification reaction, the amplified target fragment was 310 bp. Representative PCR amplification results are shown in Fig. 1. Among all of the 52 genomic DNA samples, the electrophoresis results of the PCR products show that *IGHV* gene was positively amplified in 47 tissue samples. In the other 5 tissue samples, PCR directed *IGHV* gene amplification was negative.

### 3.2 Sequence analysis of *IGHV* gene in mantle cell lymphoma paraffin tissue samples

**Figure 2:**
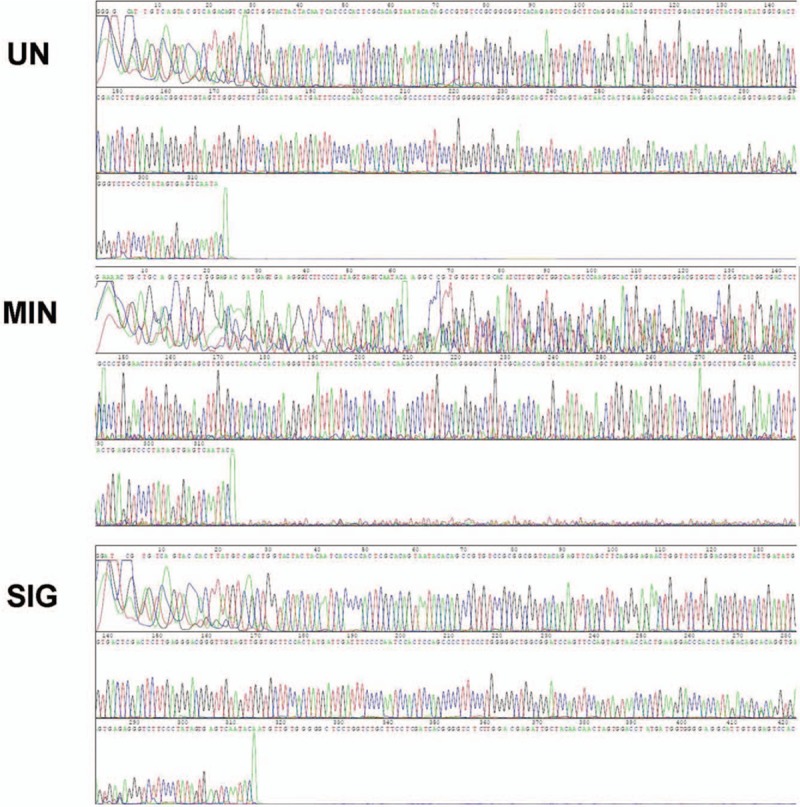
Sequence analysis of *IGHV* gene in mantle cell lymphoma paraffin tissue samples. All 47 DNA samples with positive PCR amplification of the *IGHV* gene were analyzed by DNA bi-directional sequencing. Representative sequencing results in the UN, Min, and Sig groups are shown. *IGHV* = immunoglobulin heavy chain variable region, Min = minimally mutated group, PCR = polymerase chain reaction, Sig = significantly mutated group, UN = non-mutated group.

All 47 DNA samples that showed positive PCR amplification of *IGHV* gene were subjected to DNA bi-directional sequencing and IgBLAST DNA sequence alignment analysis, according to the methods that have been previously described.^[4]^ Representative DNA bi-directional sequencing results are shown in Fig. 2. In comparison with the published *IGHV* gene germline sequences, the coincidence rate with the collected DNA samples was in the range of 94% to 100%. Among these samples, in 7 cases (15%) the coincidence rate was 100% (defined as non-mutated [UN] group); in 30 cases (64%) the coincidence rate was 97.0% to 99.9% (defined as minimally mutated [Min] group), and in 10 cases (21%) the coincidence rate was 94.0% to 96.9% (defined as significantly mutated [Sig] group). The grouping of UN, Min, and Sig was determined according to a previously published study.

### 3.3 Association analysis of *IGHV* gene mutation with the clinical outcome of mantle cell lymphomas

**Figure 3:**
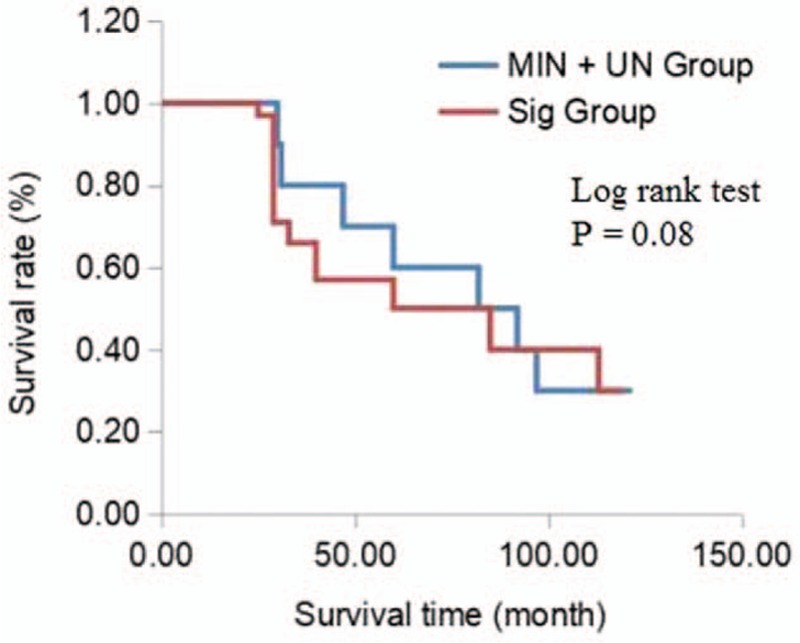
Kaplan–Meier survival curves of patients with different *IGHV* gene coincidence rates. The blue line displays the data from the MIN + UN group (37 cases; 97–100% coincident rate); the red line displays the data from the Sig group (10 cases; 94–96.9% coincident rate). The survival rate of the Sig group tends to be worse than that of the other groups, however the difference does not reach statistical significance (log-rank test, *P* = .08). *IGHV* = immunoglobulin heavy chain variable region, Min = minimally mutated group, Sig = significantly mutated group, UN = non-mutated group.

There was no significant difference in the age or sex of patients between groups (all *P* > .05, Table 1). The patients were all clinically diagnosed with MCL at stage II–IV. By comparing the clinical and histopathological data of the 3 groups, we found that the Sig group tended to show a slightly higher incidence of enlarged lymph nodes (>1 cm) and pathological stage IV than those of the other groups (Sig group: 80% and 90%; Min group: 70% and 80%, UN group: 71% and 57%, respectively) but this difference was not significant (all *P* > .05). Of the 47 patients, 41 had cyclophosphamide hydroxydaunomycin oncovin prednisolone chemotherapy. The median follow-up time after chemotherapy was 40 months and there was no statistically significant difference in the follow-up time between the groups (*P* > .05). However, the Sig group showed a lower chemotherapy response and a shorter 5-year survival time than the other groups (Sig group: 20% and 30%; Min group: 30% and 53%, UN group: 29% and 43%, respectively). Analysis of variance (ANOVA) showed that there was a significant difference in the chemotherapy response (*P* = .04) and the 5-year survival time (*P* = .03) between the 3 groups. Further survival analysis, with log-rank test, showed that there was a trend toward a worsening survival in the Sig group than in the other groups, however statistical significance was not reached (Fig. 3, log-rank test, *P* = .08).

**Table 1:**
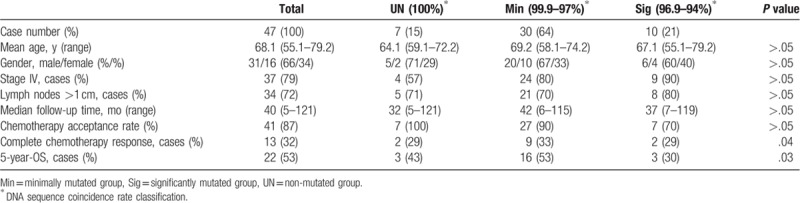
Clinical biological information of patients with MCL grouped according to *IGHV* gene mutation status.

## 4 Discussion

MCL typically has chromosome translocations such as t (11; 14) resulting in the insertion of the immunoglobulin heavy chain (IGHV) on chromosome 14q32 into oncogene cyclin D1 (CCND1) on chromosome 11q13, which is believed to be the major mechanism involved in MCL development.^[5]^ In MCL patients, *IGHV* genes undergo mutations. MCL usually presents as an aggressive process, however some cases of MCL can develop slowly.^[14–18]^ Recently, published data have indicated that the extent of *IGHV* mutation may be related to the prognosis of MCL.^[16,19,20]^ In the present study, we retrospectively explored whether the extent of *IGHV* mutations can be used to predict the clinical manifestations of MCL, such as the response to chemotherapy, the survival of patients, and the rate of disease progression.

In mouse models, mutation rate of *IGHV* gene in MCL tumor cells has been found to be related to the progression and the outcome of chemotherapy and radiotherapy.^[4]^ In the present study, using clinical MCL samples, we found that the genomic DNA of 47 cases obtained from 52 paraffin-embedded MCL tissues were positive for *IGHV* genes, and the remaining 5 cases were negative for *IGHV* gene amplification. Further histological examination demonstrated that the 5 cases that were negative for *IGHV* gene amplification were from paracancerous tissues. DNA sequencing of the PCR amplified *IGHV* gene of the positive 47 cases found that the coincidence rate of *IGHV* gene variable region sequences and the published germ line sequences ranged from 94% to 100%. Among these, 7 cases (15%) were 100% non-mutated, 30 cases (64%) were minimal mutated, and 10 cases (21%) were significantly mutated with the coincidence rate of 94.0 to 96.9. The mutation rates of *IGHV* genes that were determined in the present study were consistent with the results of a previous study.^[19]^

Previous studies have demonstrated that *IGHV* gene mutation status is associated with the prognosis of chronic lymphocytic leukemia patients after treatment with chemotherapeutics and molecular targeted drugs.^[12,13]^ Though our results showed no statistically significant correlation between the mutation rate of *IGHV* gene and the incidence of clinical stage IV and lymph node enlargement (>1 cm), we revealed that the complete response to chemotherapy and 5-year survival rate of MCL patients were negatively correlated with the degree of *IGHV* gene mutation. Furthermore, ANOVA analyses demonstrated that there was a significant difference between the 3 groups. Survival analysis, by log-rank test, further revealed that the patients with significant *IGHV* gene mutation showed a trend towards survival deterioration, although the survival rate of the 3 groups did not show a significant difference. In a previous study, multivariate analysis of *IGHV* gene status expression from 177 MCL patients suggested that *IGHV* is an independent risk factor.^[19,21]^ The failure to obtain a statistically significant difference in the patient clinic outcome depending on the level of *IGHV* mutation in our study may be due to the limited patient numbers. Nevertheless, together with previously published studies, our results support the proposal that the determination of the mutation rate or coincidence rate of *IGHV* gene variable region is valuable in judging the prognosis and therapeutic reactivity of MCL.

## 5 Conclusion

We have demonstrated that the mutation rate of the variable region of *IGHV* gene has a tendency to be correlated with the clinical stage IV, lymph node enlargement, poor chemotherapy response, and shorter 5-year survival in MCL patients. Our findings suggest the importance of the determination of the mutation rate or coincidence rate of the variable region sequences of *IGHV* gene in judging the prognosis and therapeutic reactivity of MCL after definitive clinical diagnosis. Nevertheless, it should be noted that the number of samples involved in this study was very limited, which may result in incomplete agreement between the results of the 2 statistical analysis methods. Future studies with a large cohort of MCL patients throughout multiple centers are needed.

## Author contributions

**Conceptualization:** Xianqian Li.

**Data curation:** Bin Li.

**Formal analysis:** Ning Wu, Bin Li.

**Funding acquisition:** Xianqian Li, Ning Wu, Bin Li.

**Investigation:** Bin Li.

**Methodology:** Ning Wu.

**Project administration:** Ning Wu, Bin Li.

**Resources:** Ning Wu.

**Supervision:** Xianqian Li.

**Validation:** Xianqian Li, Bin Li.
